# Autologous Fat Grafting Reduces Pain in Irradiated Breast: A Review of Our Experience

**DOI:** 10.1155/2016/2527349

**Published:** 2015-12-29

**Authors:** Fabio Caviggioli, Luca Maione, Francesco Klinger, Andrea Lisa, Marco Klinger

**Affiliations:** ^1^Reconstructive and Aesthetic Plastic Surgery School, University of Milan, MultiMedica Holding S.p.A., Plastic Surgery Unit, Via Milanese 300, Sesto San Giovanni, 20099 Milan, Italy; ^2^Reconstructive and Aesthetic Plastic Surgery School, Department of Medical Biotechnology and Translational Medicine (BIOMETRA), University of Milan, Plastic Surgery Unit, Humanitas Research Hospital, Via Alessandro Manzoni 56, Rozzano, 20089 Milan, Italy

## Abstract

*Introduction*. Pain syndromes affect women after conservative and radical breast oncological procedures. Radiation therapy influences their development. We report autologous fat grafting therapeutical role in treating chronic pain in irradiated patients. *Materials and Methods*. From February 2006 to November 2014, we collect a total of 209 patients who meet the definition of “Postmastectomy Pain Syndrome” (PMPS) and had undergone mastectomy with axillary dissection (113 patients) or quadrantectomy (96 patients). Both procedures were followed by radiotherapy. We performed fat grafting following Coleman's procedure. Mean amount of adipose tissue injected was 52 cc (±8.9 cc) per breast. Seventy-eight in 209 patients were not treated surgically and were considered as control group. Data were gathered through preoperative and postoperative VAS questionnaires; analgesic drug intake was recorded. *Results*. The follow-up was at 12 months (range 11.7–13.5 months). In 120 treated patients we detected pain decrease (mean ± SD point reduction, 3.19 ± 2.86). Forty-eight in 59 patients stopped their analgesic drug therapy. Controls reported a mean ± SD decrease of pain of 1.14 ± 2.72. Results showed that pain decreased significantly in patients treated (*p* < 0.005, *Wilcoxon rank-sum test*). *Conclusion*. Our 8-year experience confirms fat grafting effectiveness in decreasing neuropathic pain.

## 1. Introduction

Chronic pain affects from 25% to 60% of women submitted to breast surgery, both mastectomy and conservative procedures for oncological reasons, and represents an important clinical problem involving intra- and postoperative factors [1].

This condition, also named Postmastectomy Pain Syndrome (PMPS), is situated in the anterior side of the thorax, in the axilla, and/or in the upper half of the arm and lasts more than 3 months after mastectomy or quadrantectomy surgical procedures [2].

Several risk factors have been advocated to explain the development of PMPS [3] such as axillary lymph node dissection [4], chemotherapy agents [5], and postsurgical complications such as infection, seroma, or hematoma.

Moreover, BMI, age, and physiological status could also be a predictive factor in postsurgical pain [6].

Beyond all these multifactorial risk factors, radiotherapy strongly influences the development of PMPS as already noted by Tasmuth et al. [7].

Medications commonly used to treat nociceptive pain, such as opioids, have proved to be ineffective for neuropathic pain and a chronic analgesic therapy is burdened with complications and high rate of drop-off.

In our experience, autologous fat graft obtained following Coleman's technique has proven to be an efficient and safe procedure in treating the clinical field of breast reconstructive surgery, providing an important tool for correction of breast size and shape.

In 2006, our group started treating patients submitted to mastectomy with axillary dissection and radiotherapy [8] demonstrating the capability of lipostructure to relieve chronic pain. We subsequently extended its indications to patients submitted to quadrantectomy followed by radiotherapy [9].

In both groups we observed a significant decrease in pain after procedure and a reduction of intake of analgesic drug therapy.

The present review aims to report our experience in treating chronic pain in irradiated breasts collected after an 8-year experience confirming autologous fat grafting role in improving pain control in patients affected by PMPS.

## 2. Patients and Methods

From February 2006 to November 2014, a total of 209 patients who had undergone mastectomy with axillary dissection (113 patients) or quadrantectomy (96 patients) came to our Institution.

Both procedures were followed by radiotherapy for oncological reasons.

We selected patients who presented severe scar retraction, radiodystrophy, and chronic pain meeting the definition of “Postmastectomy Pain Syndrome.”

All patients enlisted had a normal follow-up with no complications, such as dehiscence, infection, or scar anomalies.

In our study population, we performed autologous fat grafting following Coleman's procedure. After routine preoperative examination and clinical assessment, patients underwent liposuction of the subumbilical area under sedation and local anesthesia. The adipose tissue was harvested from abdominal subcutaneous fat which is an easy accessible and abundant reservoir.

The fat graft was injected using 18-gauge angiographic needle with a snap-on wing (Cordis, a Johnson & Johnson Company, N. V, Roden, Netherlands) at the dermohypodermal junction in the painful and radiodystrophic scar areas. The mean amount of adipose tissue injected was 52 cc (±8.9 cc) per breast. In particular, in patients submitted to mastectomy, mean amount of adipose tissue was 55 cc (±9.3 cc) per breast, while in patients submitted to quadrantectomy mean value was 39 cc per breast (±5.4 cc).

Seventy-eight in 209 patients with Postmastectomy Pain Syndrome (41 submitted to mastectomy and 37 to quadrantectomy) not treated surgically were considered as a control group for statistical analysis.

Patients were assigned to control group due to their refusal to be submitted to surgical procedure, although they present comparable VAS scores.

During the first visit, a trained research assistant explained the purpose and methods of this study to each eligible patient; patients who were willing to participate signed an informed agreement.

Data were gathered through preoperative and postoperative questionnaires.

Before undergoing the surgical procedure, patients were required to score their spontaneous pain using a visual analogue scale ranging from 0 to 10; analgesic drug intake was also recorded.

During follow-up patients were required to fill out the same questionnaire and possible modification in analgesic intake was also accurately recorded.

## 3. Results 

We collected a total of 209 patients; 113 patients were submitted to mastectomy with axillary dissection, while 96 patients were submitted to quadrantectomy.

In our population we lost a total of nineteen patients in the follow-up (11 were in case group and 9 were submitted to mastectomy and 2 were submitted to quadrantectomy, while 8 were controls, 6 who underwent previous mastectomy and 2 who underwent quadrantectomy).

In 120 treated patients we observed a mean VAS before fat grafting of 7.2 (SD ± 2.1) and a value of 3.3 (SD ± 3.1) after treatment so that we detected a mean pain decrease of 3.19 (SD ± 2.86).

This effect has been observed since two weeks from the procedure.

In the treated group a total of 48 patients stopped their analgesic drug therapy.

In this group mean VAS before fat grafting was 7.7 (±2.7) and 3.4 (±2.4) after autologous fat grafting; thus the mean pain decrease was 4.23 (SD ± 2.14).

In the group of patients that continued analgesic therapy mean VAS before treatment was 7.9 (±1.9), while after treatment mean value was 4.2 (±2.3) with a mean pain decrease of 1.15 (SD ± 2.79).

Control group at first outpatient visit showed a mean VAS of 6.9 (±2.2), while at follow-up visit they showed a mean VAS of 5.8 (±1.9) reporting a mean pain decrease of 1.14 (SD ± 2.72).

Statistical analysis of the difference between VAS values was made before and after treatment in case patients and at first outpatient visit and at follow-up for controls. In both groups the follow-up was at 12 months (range 11.7–13.5 months).

Wilcoxon rank-sum test was employed with a level of significance fixed at *p* < 0.005.

Results showed that pain decreased significantly in patients with Postmastectomy Pain Syndrome treated with autologous fat tissue grafting (*p* < 0.005), while in control patients no statistical difference was found (*p* > 0.05).

In addition to that we compared VAS values in case patients before treatment with control patients at first outpatient visit. Eventually we compared again VAS values in case patients after treatment with control patients at follow-up visit. Mann and Whitney test was employed with a level of significance fixed at *p* < 0.005. While in the first comparison no statistically significant difference was noted (*p* > 0.05), in the latter a statistical significant difference was observed (*p* < 0.005). By performing this test selection bias was excluded. Results are listed in Table 1.

## 4. Discussion

Persistent pain as a consequence of surgical treatment has been established for several common surgical procedures and represents a clinical problem of great magnitude.

Postmastectomy Pain Syndrome was firstly reported in the 1970s by Wood [10]. Subsequently the International Association for the Study of Pain defined it as a chronic pain condition with characteristics resembling neuropathic pain.

In patients submitted to quadrantectomy or mastectomy, it presents common features and for this reason it is considered as a unique pathological condition characterized by a dull, burning, and aching sensation exacerbated by movement of the shoulder girdle [11].

Women affected by PMPS may present signs and symptoms such as neck pain, shoulder pain, reduced mobility, and bad body image, demonstrating a multidimensional character that affects psychological and physical aspects of the patient's life [12].

Though the exact pathogenesis of PMPS remains unclear, many etiological theories have been postulated.

During the surgical procedure, dissection of the intercostobrachial nerve or damage of axillary nerve pathways could favor development of chronic pain after mastectomy or quadrantectomy. For this reason, chronic pain has a greater rate of occurrence in patients who underwent axillary lymph node dissection [4].

Chemotherapy agents, such as taxanes, platinum agents, and vinca alkaloids, are neurotoxic, while endocrine therapy may induce musculoskeletal pain and arthralgia [5].

Another possible explanation seems to be related to the scarring process which leads to fibrosis, strong adherence to the deeper muscular layer, and possible nervous entrapment which would represent a continuous trigger of nerve excitation. For this reason, postsurgical complications [13, 14] such as infection, seroma, and hematoma may be potential sources of the development of persistent pain altering physiological scarring.

Radiation therapy represents a major risk factor for developing of PMPS since it leads to an inflammatory reaction accompanied by increased production of proinflammatory cytokines, such as IL-1, IL-6, TNF-alfa, and TGF-beta, and chemokines such as IL-8 and eotaxin. This inflammatory reaction can induce peripheral and central sensitization with a failed nociception system leading to pain augmentation. In addition to that, radiotherapy stimulates local fibrosis which could result in a strong adherence to the deeper muscular layer, sustaining a painful syndrome.

Actually, the mainstay of treatment for PMPS as any form of neuropathic pain is pharmacological, including the use of antidepressants [15], antiepileptics, and topical anesthetics, such as lidocaine patches and opioids. Since a pharmacologic therapy lasting for a long period is poorly tolerated, nonpharmacological treatments have been proposed including psychological approaches, physical therapy, interventional therapy, spinal cord stimulation, and surgical procedures. Nevertheless, neuropathic pain remains difficult to treat, and a combination of therapies may be more effective than monotherapy.

Our group firstly proposed autologous fat graft as an effective complementary approach to relieve PMPS. Moving from the experience of Rigotti et al. [16] who treated radiotherapy-induced tissue damage, several studies focused on the capability of fat graft to induce scar tissue architectural remodelling and regeneration with neoangiogenesis.

In our previous researches, histological proofs [17–19] have demonstrated how fat grafting could be responsible for scar remodelling inducing release of fibrotic tissue with nerve liberation and loose connective regeneration, leading to increased scar softness.

We have also hypothesized that autologous fat graft could induce molecular changes in the microenvironment of posttraumatic scar [20], which is hostile to regeneration of the nervous system because of intrinsic inhibitory factors expressed by extracellular matrix, as shown by experimental model of spinal cord injuries [21], determining pain control as described in patients affected by Arnold's Neuralgia [22, 23].

It is possible to hypothesize that fat grafting in PMPS induces analgesia by inhibition of inflammation. It is relevant to notice that mesenchymal stem cells could also downregulate radiation therapy induced inflammatory response as they inhibit production and release of proinflammatory cytokines, which are the main mediators of radio-induced tissue damage.

In the present review we report our 8-year experience in treating Postmastectomy Pain Syndrome with autologous fat grafting in patients submitted to mastectomy with axillary dissection and quadrantectomy and radiotherapy.

We considered patients affected as a unique study population as they all meet the definition of Postmastectomy Pain Syndrome.

The main limitation of this study is the fact that we did not consider the placebo effect to give further strength to our protocol.

In addition to that our research's lack of data regarding patients' age, BMI, and estrogen status as increasing evidence seems to correlate these variables to clinical outcome [24].

Comparing data with a wide study population of 120 patients, to our knowledge the largest study population presented in a scientific report about this condition, we confirm autologous fat grafting's promising therapeutic role in the treatment of Postmastectomy Pain Syndrome.

## 5. Conclusions

Fat graft has already proven its effectiveness in terms of pain decrease in the treatment of different forms of neuropathic pain as a result of its effect of scar entrapment release and anatomical remodeling.

Because of its safety, efficacy, and optimal tolerability, we support its adoption as a standard procedure in each Breast Unit in patients who report chronic pain after breast surgery especially if previously submitted to radiotherapy.

## Figures and Tables

**Table 1:** Study population description. We differentiate between patients who report PMPS after mastectomy and patients who report PMPS after quadrantectomy. Analgesic drug intake was recorded for both groups. We report mean VAS values before and after treatment or at follow-up for controls; mean and median VAS values decrease together with ranges. Analysis of pain before and after treatment in both case and control patients was performed by means of Wilcoxon test; *p* value significance was fixed at < 0.005. We obtain *p* < 0.005 in cases and *p* > 0.05 in controls. Moreover, by means of Mann and Whitney, we compare VAS values in case patients before treatment with controls (*p* > 0.05) and in case patients after treatment with controls test (*p* < 0.005).

	PMPS after mastectomy	Mean VAS before treatment	PMPS after quadrantectomy	Mean VAS after treatment or at follow-up	Mean VAS decrease	Median VAS decrease	Range VAS decrease
Treated	63 (120 total treated patients)	7.2 (±2.1)	57 (120 total treated patients)	3.3 (±3.1)	3.19 (±2.86)	2.63	−2.1–9.6

Control	35 (70 total control patients)	6.9 (±2.2)	35 (70 total control patients)	5.8 (±1.9)	1.14 (±2.72)	1.09	−4.2–6.3

Stop pharmacologic therapy	28 (34 total patients who assumed therapy)	7.7 (±2.7)	20 (25 total patients who assumed therapy)	3.4 (±2.4)	4.23 (±2.14)	4.86	−1.6–9.3

Continue pharmacologic therapy	6 (34 total patients who assumed therapy)	7.9 (±1.9)	5 (25 total patients who assumed therapy)	4.2 (±2.3)	1.15 (±2.79)	1.0	2.2–2.5
